# *MiR-5195-3p* functions as a tumor suppressor in prostate cancer via targeting *CCNL1*

**DOI:** 10.1186/s11658-022-00326-8

**Published:** 2022-03-08

**Authors:** Xing Zeng, Zhiquan Hu, Yuanqing Shen, Xian Wei, Jiahua Gan, Zheng Liu

**Affiliations:** Department of Urology, Tongji Hospital, Tongji Medical College, Huazhong University of Science and Technology, No. 1095 Jiefang Ave, Wuhan, 430030 Hubei China

**Keywords:** Prostate cancer, *miR-5195-3p*, *CCNL1*, Proliferation

## Abstract

**Background:**

Accumulating evidence indicates that *miR-5195-3p* exerts tumor-suppressive roles in several tumors. However, the clinical significance and biological function of *miR-5195-3p* in prostate cancer (PCa) have not been reported yet.

**Methods:**

The expression levels of *miR-5195-3p* and *Cyclin L1 (CCNL1)* were determined using quantitative real-time PCR in clinical specimens and cell lines. The clinical significance of *miR-5195-3p* in patients with PCa was evaluated using Kaplan–Meier survival analysis and Cox regression models. Cell proliferation and cell cycle distribution were measured by CCK-8 assay and flow cytometry, respectively. The association between *miR-5195-3p* and *CCNL1* was analyzed by luciferase reporter assay.

**Results:**

*MiR-5195-3p* expression levels were significantly downregulated in 69 paired PCa tissues compared with matched adjacent normal tissues. The decreased *miR-5195-3p* expression was associated with Gleason score and TNM stage, as well as worse survival prognosis. The in vitro experiments showed that *miR-5195-3p* overexpression suppressed the proliferation and cell cycle G1/S transition in PC-3 and DU145 cells. Elevated *miR-5195-3p* abundance obviously impaired tumor formation in vivo using PC-3 xenografts. Mechanistically, *CCNL1* was a direct target of *miR-5195-3p* in PCa cells, which was inversely correlated with *miR-5195-3p* in PCa tissues. Importantly, *CCNL1* knockdown imitated, while overexpression reversed, the effects of *miR-5195-3p* overexpression on PCa cell proliferation and cell cycle G1/S transition.

**Conclusions:**

Our data suggest that *miR-5195-3p* functions as a tumor suppressor by targeting *CCNL1* in PCa.

## Background

Prostate cancer (PCa), as the most frequently diagnosed male malignancy, has been the leading cause of tumor-related deaths worldwide, with its pathological and clinical heterogeneity [1, 2]. It is estimated that there are more than 220,000 new cases of PCa and over 30,000 death per year in the USA [3]. Despite great improvement in the outcomes of PCa at early stage by early prostate-specific antigen (PSA) testing, surgical resection, and androgen deprivation therapy [4, 5], the prognosis is still poor for patients at advanced stage, especially for those with emergence of castration-resistant PCa [6]. Therefore, elucidation of the molecular mechanisms underlying the initiation and progression of PCa is urgently needed to establish new therapeutic targets for PCa treatment.

MicroRNAs (miRNAs/miRs) are a group of small, noncoding, and single-stranded RNAs approximately 18–22 nucleotides in length, which can regulate multiple physiological processes by selectively inhibiting the downstream target mRNAs via binding to their 3′-untranslated region (3′-UTR) [7, 8]. In recent years, growing evidence has suggested that certain aberrantly expressed miRNAs can act as either oncogenes or tumor suppressors, thereby affecting the pathogenesis of PCa. For example, overexpression of *miR-139* inhibited the cell growth and migration in PCa cells [9]. Overexpression of *miR‑589‑5p* inhibited cell viability, migration, and invasion in PCa cells [10]. On the contrary, some oncogenic factors, including *miR-410-3p* [11], *miR-153* [12], and *miR-191* [13], have been identified as carcinogenic factors that predict poor prognosis and promote the proliferation of PCa cells. Notably, *miR-5195-3p*, a relatively poorly studied miRNA, has been shown to participate in cell biological processes that regulate the progression of tumors, including non-small cell lung cancer [14], glioma [15], osteosarcoma [16], and bladder cancer [17]. However, there are limited studies on the clinical significance and biological function of *miR-5195-3p* in PCa.

*Cyclin L1* (*CCNL1* also termed Ania-6a), localized in the chromosomal 3q25 region, codes for a putative key regulator of pre-mRNA processing and is involved in G1/S transition during the cell cycle [18]. Interestingly, *CCNL1* has been illustrated as a potential target of therapeutic interventions implicated in carcinogenesis [19]. As reported by Redon et al. [20] and Sticht et al. [21], *CCNL1* was overexpressed and amplified in human head and neck squamous cell carcinoma. Moreover, *CCNL1* has been demonstrated to be a direct target gene of *miR-199b-5p* and be involved in *miR-199b-5p* suppressing cell proliferation and inducing cell cycle arrest and cell apoptosis in Ewing’s sarcoma [22]. According to the online software program TargetScan 7.1 prediction that *CCNL1* was a potential target of *miR-5195-3p*, we speculated that *miR-5195-3p* might play an important role in PCa tumorigenesis by targeting *CCNL1* via affecting cell cycle progression.

Therefore, we first investigated the expression pattern and clinical significance of *miR-5195-3p* in PCa tissues. Next, we conducted a series of in vitro and in vivo functional experiments to observe the regulatory roles of *miR-5195-3p* in cell proliferation and tumor growth. Furthermore, we validated the association between *miR-5195-3p* and *CCNL1* in PCa. This research will enhance our understanding of PCa biology and provide new insights into molecular therapy for PCa treatment.

## Materials and methods

### Patients and tissue samples

In total, 69 pairs of tumor tissues and matched adjacent normal tissues were obtained from patients with PCa who underwent radical prostatectomy at Tongji Hospital, Tongji Medical College, Huazhong University of Science and Technology (Hubei, China) between March 2016 and September 2018. Prior to radical prostatectomy, all patients were confirmed not to receive chemotherapy, radiotherapy, or androgen deprivation therapies. All tissue specimens were immediately stored at −80 °C until further use. The basic clinicopathological characteristics of all patients with PCa, including age, Gleason score, and TNM stage, are summarized in Table 1. All enrolled patients underwent 5-year follow-up through telephone. Written informed consent was signed by all patients, and the present study was approved by the ethics committee of Tongji Hospital, Tongji Medical College, Huazhong University of Science and Technology (approval number TMCU-87DG; 2016.3.12; Hubei, China).

**Table 1:** Relationship between miR-5195-3p expression and clinicopathological characteristics of patients with prostate cancer

Characteristic	Case (*n* = 69)	miR-5195-3p expression		*p*-Value
		Low (*n* = 35)	High (*n* = 34)	(chi-squared test)
Age (year)				0.733
< 65	21	10	11	
≥ 65	48	25	23	
Preoperative PSA (ng/ml)				0.368
< 10	43	20	23	
≥ 10	26	15	11	
Gleason score				0.001*
< 7	51	20	31	
≥ 7	18	15	3	
TNM stage				0.006*
I/II	46	18	28	
III/IV	23	17	6	
Metastasis				0.118
No	39	23	16	
Yes	30	12	18	

*p < 0.05

### Cell culture

Human PCa cell lines (PC-3, 22RV1, DU145, and LNCaP) and a nontransformed but immortalized prostate cell line RWPE-1 were purchased from the American Type Culture Collection (Manassas, VA, USA). All cell lines were cultured in RPMI-1640 (Gibco; Thermo Fisher Scientific) supplemented with 10% fetal bovine serum (Gibco) in a humidified incubator containing 5% CO_2_ at 37 °C.

### Cell transfection

*MiR-5195-3p* mimics, scrambled miRNA (miR-NC), smaller interfering RNA against *CCNL1* (si-CCNL1), negative control (si-NC), the overexpression plasmid of pcDNA3.1-CCNL1, and empty vector pcDNA3.1 were synthesized from Shanghai GenePharma. For cell transfection, PC-3 and DU145 cells were seeded into six-well plates and transfected with the above oligonucleotides using Lipofectamine 2000 (Thermo Fisher Scientific) according to the manufacturer’s instructions.

### Quantitative real-time PCR

Total RNA was extracted using mirVana miRNA isolation kit (Life Technologies; Thermo Fisher Scientific) for miRNA and RNeasy mini kit (Qiagen, Valencia, CA, USA) for mRNA. The synthesis of complementary DNA was performed using miScript II RT kit (Applied Biosystems, CA) for miRNA and superscript VILO cDNA kit (Thermo Fisher Scientific) for mRNA according to the manufacturer’s instructions. Quantitative real-time PCR was performed using miScript SYBR Green PCR kit (Qiagen) for *miR-5195-3p* or SYBR Green PCR kit (Applied Biosystems, CA) for *CCNL1* mRNA levels with the specific primer sequences synthesized by Sangon Biotech (Shanghai). Each experiment was performed in triplicate, and relative abundance was normalized to *U6* for *miR-5195-3p* or *GADPH* for *CCNL1* mRNA by the 2^−ΔΔCT^ method. The primer sequences used were as follows: *CCNL1*, forward 5′-GGAAAAAGGACTCCAAGCCC-3′ and reverse 5′-GCTGCAAGGTAGATGCAAGC-3′; *GAPDH*, forward 5′-GGTGAAGGTCGGAGTCAACG-3′ and reverse 5′-GCATCGCCCCACTTGATTTT-3′.

### Cell proliferation assay

Transfected PCa cells at a density of 3 × 10^4^ cells per well in technical triplicates were seeded in six-well plates and cultured for 24, 48, and 72 h, respectively. At each timepoint, cells in each well were incubated with 10 µl of CCK-8 solution (Sigma-Aldrich) for 2 h. Afterwards, the absorbance at a wavelength of 450 nm was measured in each well using a microplate reader.

### Cell cycle analysis

Transfected PCa cells at a density 4 × 10^5^ cells per well were seeded in six-well plates and incubated for 48 h. Subsequently, cells were washed with PBS and fixed with cold 70% ethanol overnight, followed by incubation with 0.1 mg/ml propidium iodide (Sigma-Aldrich) for 30 min in the dark. Next, the cells were analyzed by flow cytometry (BD Biosciences, Franklin Lakes, NJ, USA) with FlowJo software (Version 10.0.4; FlowJo LLC).

### Luciferase reporter assay

According to the putative binding sites predicted for *miR-5195-3p* with the 3′‐UTR of *CCNL1* by the online software program TargetScan 7.1 (http://www.targetscan.org), we performed luciferase reporter assay to validate the above prediction. In brief, the fragments of *CCNL1* 3′‐UTR containing either putative *miR-5195-3p* seed sequence or corresponding mutant (MUT) sites using QuickChange Site-Direct Mutagenesis Kit (Stratagene) were subcloned into psiCHECK‐2 vector (Promega, USA) to obtain the reporter plasmids of *CCNL1*-wild type (WT) and *CCNL1*-MUT, respectively. Then, PC-3 or DU145 cells were plated in 24-well plates and co-transfected with 1 μg reporter plasmid *CCNL1*-WT or *CCNL1*-MUT together with 30 nM *miR-5195-3p* mimics or miR-NC for 48 h. Relative luciferase activity was determined using a Dual‐Luciferase Reporter Assay System (Promega).

### Tumor xenograft formation

For the tumorigenicity assay, miR-NC or miR-5195-3p mimics stably transfected overexpression 1.8 × 10^6^ PC-3 cell suspension was injected subcutaneously into the right flank of 4–5-week-old BALB/c nude mice (Shanghai Laboratory Animal Research Center, Shanghai, China) with five mice in each group. Mice were monitored every 5 days, and the tumor length/width was measured using calipers. Tumor volume was calculated using the modified ellipsoid formula: volume = 1/2 (length × width^2^). At the end of 30 days, all the mice were killed by cervical dislocation. Then, the tumor weight was measured and tumor tissues were harvested for analyzing the expression levels of *miR-5195-3p*, as well as protein levels of Cyclin L1, CDK4, and Cyclin D1. All animal experiments were performed in accordance with the Huazhong University of Science and Technology Research Institute Animal Care Committee guidelines (approval number HUST-58A; 2017.6.23; Hubei, China).

### Western blot analysis

Total protein samples were extracted from cell lines or tumor tissues with RIPA lysis buffer (Thermo Fisher Scientific), and protein concentration was analyzed using a BCA Protein Assay Kit (Pierce, Rockford, IL, USA) according to the manufacturer’s instructions. Then, equal amount of protein sample (30 µg) was subjected to electrophoresis using sodium dodecyl sulfate polyacrylamide gels (SDS‐PAGE), which was subsequently transferred onto PVDF membranes. Next, the membranes were blocked with 5% nonfat dried milk in TBST for 2 h and incubated with primary antibodies against Cyclin L1 (1:1000, no. PA5-36070, Thermo Fisher Scientific), CDK4 (1:1000, ab226474, Abcam), Cyclin D1 (1:5000, ab226977, Abcam), and GAPDH (1:1,000; ab37168, Abcam) overnight at 4 °C. Subsequently, the membranes were incubated with horseradish-peroxidase-linked secondary antibodies (1:5000; cat. no. SC-2054; Santa Cruz Biotechnology) for 2 h at room temperature. Finally, the protein bands were visualized by a chemiluminescence detection kit (ECL, Millipore, USA).

### Statistical analysis

The GraphPad Prism 6.0 software (National Institutes of Health, Bethesda, MD, USA) was used to perform all statistical analysis. The association between *miR-5195-3p* expression and PCa clinicopathologic characteristics was assessed by chi-squared test. The Kaplan–Meier method was used to generate survival curves. Univariate and multivariate Cox regression models were constructed to estimate the hazard ratios (HRs) of independent factors affecting the overall survival in patients with PCa. The Spearman’s correlation coefficient was used to analyze the association between *miR-5195-3p* expression and *CCNL1* expression in PCa tissues. All the quantitative data were expressed as mean ± standard deviation (SD) of at least three experimental replicates. The differences among groups were analyzed using either the one-way ANOVA or the Student’s *t*-test. Statistical significance was defined as *p*-value less than 0.05.

## Results

### *MiR-5195-3p* was downregulated in PCa tissues, which was correlated with cancer progression

**Fig. 1:**
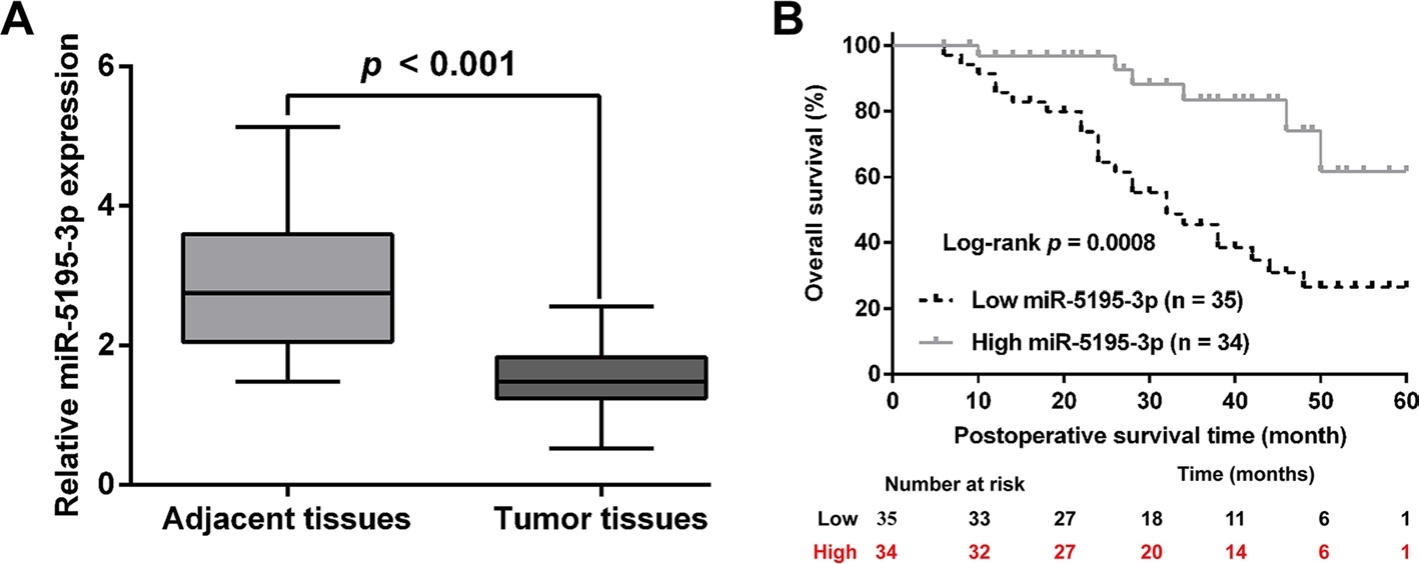
*MiR-5195-3p* was downregulated in PCa tissues and correlated with overall survival. **A** *MiR-5195-3p* expression in 69 paired HCC and the matched adjacent normal tissue samples was measured by quantitative real-time PCR. **B** The correlation between *miR-5195-3p* expression and overall survival was analyzed with the Kaplan–Meier method. The *p*-value was obtained using the log-rank test

We first performed quantitative real-time PCR analysis to determine the expression level of *miR-5195-3p* in 69 pairs of human PCa and adjacent tissues. As shown in Fig. 1A, *miR-5195-3p* expression was significantly lower in PCa tissues than in adjacent normal tissues. We then sought to explore the association between *miR-5195-3p* expression and clinical characteristics. According to the median value of *miR-5195-3p* expression, 69 patients were classified into low *miR-5195-3p* expression group (*n* = 35) and high *miR-5195-3p* expression group (*n* = 34). As listed in Table 1, *miR-5195-3p* expression was significantly associated with Gleason score and TNM stage. We further explored whether *miR-5195-3p* expression was associated with the prognosis of patients with PCa. Kaplan–Meier survival analysis revealed that patients with PCa with high *miR-5195-3p* expression had longer overall survival than those with low *miR-5195-3p* expression (Fig. 1B). We also performed univariate and multivariate Cox regression analyses of prognostic indicators using collected clinical specimens (Table 2). Univariate analysis indicated that low *miR-5195-3p* expression (HR 1.759, *p* = 0.014), Gleason score > 7 (HR 1.432, *p* = 0.023), and TNM stage (HR 2.312, *p* = 0.005) were independent risk factors for prognosis of patients with PCa. Multivariate analysis suggested that TNM stage (HR 2.542, *p* = 0.024) and low *miR-5195-3p* expression (HR 2.132, *p* = 0.007) were the hazard factors predicting overall survival in patients with PCa.

**Table 2:** Univariate and multivariate analysis for overall survival in patients with prostate cancer

Characteristic	Univariate analysis		Multivariate analysis	
	HR (95% CI)	*p* value	HR (95% CI)	*p* value
Age	0.895 (0.563–1.498)	0.754	–	–
Preoperative PSA (ng/ml)	2.145 (1.284–3.315)	0.415	–	–
Gleason score	1.432 (0.895–2.546)	0.023*	1.365 (0.968–2.584)	0.056
TNM stage	2.312 (1.204–2.978)	0.005*	2.542 (1.432–3.142)	0.024*
Metastasis	3.142 (2.142–4.321)	0.064	–	–
MiR-5195-3p expression	1.759 (1.006–2.153)	0.014*	2.132 (1.354–2.856)	0.007*

*p < 0.05

### MiR-5195-3p overexpression suppressed PCa cell proliferation and cell cycle G1/S transition in vitro

**Fig. 2:**
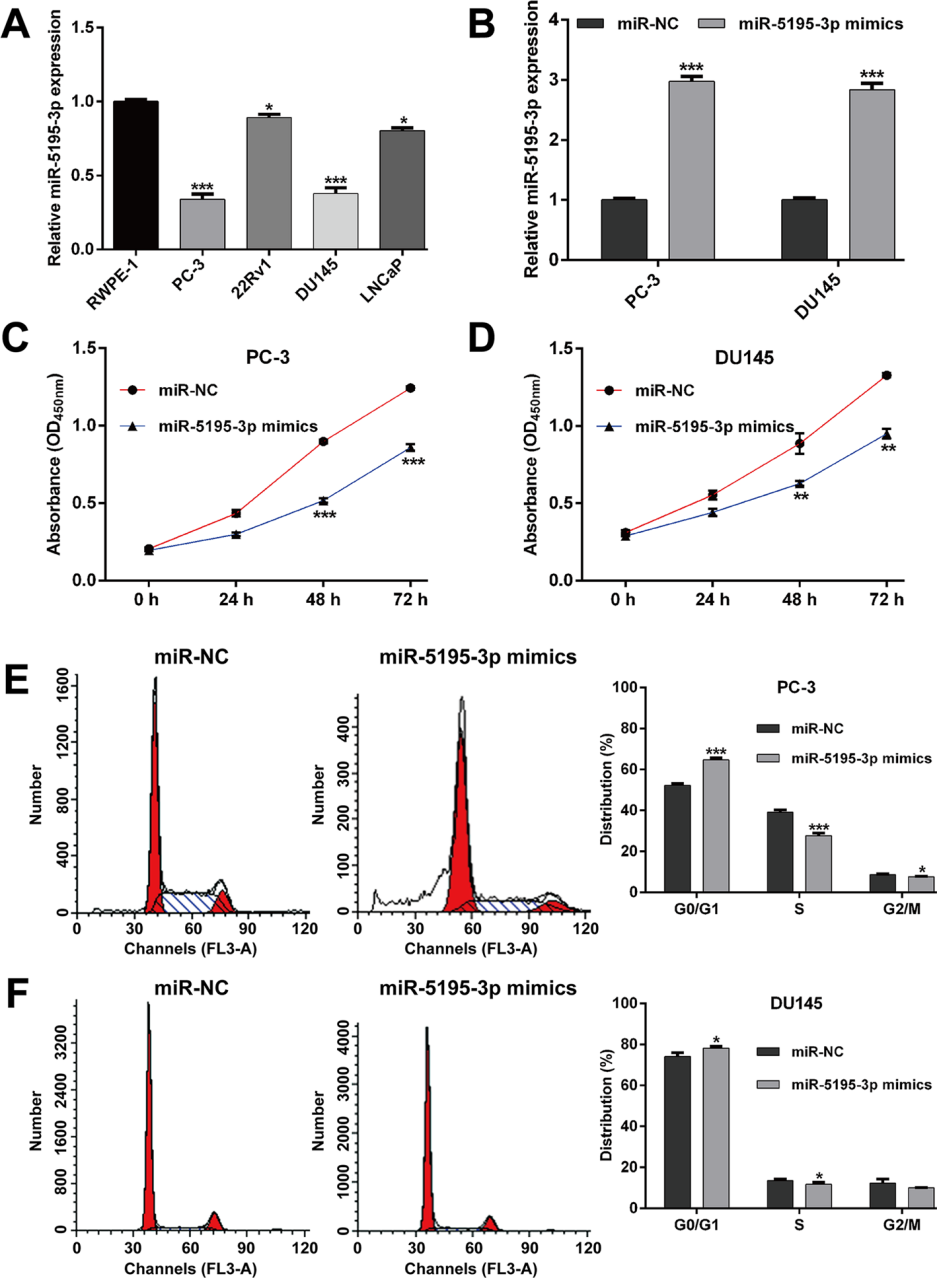
Effects of *miR-5195-3p* overexpression on PCa cell proliferation and cell cycle progression. **A** *MiR-5195-3p* expression in PCa cell lines (PC-3, 22RV1, DU145, and LNCaP) and a nontransformed but immortalized prostate cell line RWPE-1 was measured by quantitative real-time PCR. **B** PC-3 and DU145 cells were transfected with the *miR-5195-3p* mimics or miR-NC. *MiR-5195-3p* expression in PC-3 and DU145 cells was detected by quantitative real-time PCR. **C**–**D** Cell proliferation was tested with CCK-8. *MiR-5195-3p* overexpression significantly inhibited the proliferation of PC-3 and DU145 cells. **E**–**F** Cell cycle distribution was determined by PI staining and flow cytometry analysis in PC-3 and DU145 cells. Data are presented as mean ± standard deviation. *p < 0.05, **p < 0.01, ***p < 0.001 compared with miR-NC group. *PI* propidium iodide; *NC* negative control

Subsequently, the expression level of *miR-5195-3p* was assessed in several PCa cell lines. Consistently, *miR-5195-3p* expression levels were found to be significantly decreased in PCa cell lines (PC-3, 22RV1, DU145, and LNCaP) compared with a nontransformed but immortalized prostate cell line RWPE-1 (Fig. 2A). To test the biological function of *miR-5195-3p* in PCa in vitro, PC-3 and DU145 cells were transfected with *miR-5195-3p* mimics or miR-NC. As shown in Fig. 2B, *miR-5195-3p* expression was significantly elevated in both PC-3 and DU145 cells after *miR-5195-3p* mimics transfection compared with miR-NC transfection. Next, we performed gain-of-function assays in the above constructed *miR-5195-3p* overexpression cell lines. The results from CCK-8 assay showed that the cell growth curves were remarkably suppressed in PC-3 (Fig. 2C) and DU145 (Fig. 2D) cells after *miR-5195-3p* overexpression, especially at 48 and 72 h, respectively. Considering uncontrolled cell proliferation was correlated with cell cycle progression, we further analyzed the effects of *miR-5195-3p* overexpression on cell cycle distribution. The results from flow cytometry analysis illustrated that the percentage of cells at G0/G1 phase (64.71% ± 1.04% versus 52.27% ± 0.83%, p < 0.001) was significantly increased, while cells at S phase (27.68% ± 1.32% versus 39.09% ± 1.17%, p < 0.001) and G2/M phase (7.61% ± 0.28% versus 8.64% ± 0.34%, *p* < 0.05) were decreased in *miR-5195-3p* mimics group compared with miR-NC group in PC-3 cells (Fig. 2E). Similarly, we observed that *miR-5195-3p* overexpression caused a significant increase in the proportion of cells at G0/G1 phase and decrease in cells at S phase in DU145 cells (Fig. 2F). These findings indicated that *miR-5195-3p* overexpression inhibited the proliferation and induced G0/G1 arrest in PCa cells.

### *CCNL1 *is a direct target of *miR-5195-3p* in PCa

**Fig. 3:**
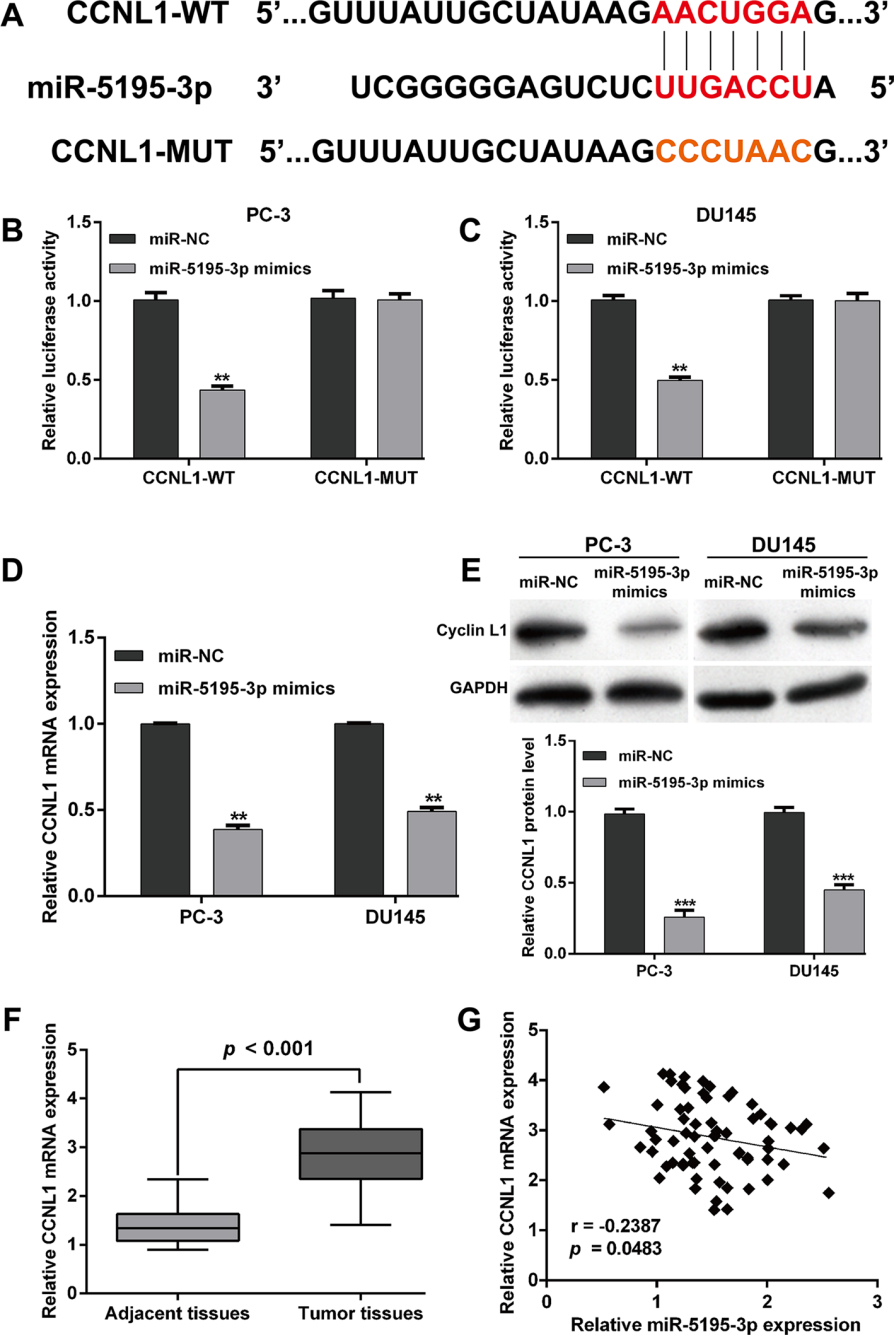
*CCNL1* was a direct target of *miR-5195-3p* in PCa. **A** The sequences of the putative *miR-5195-3p* binding sites in the wild-type and mutant *CCNL1* 3′-UTR. **B**–**C** Luciferase reporter plasmids carrying the *CCNL1* wild-type 3′-UTR (CCNL1-WT) or CCNL1 mutant 3′-UTR (CCNL1-MUT) were transfected into PC-3 and DU145 cells with *miR-5195-3p* mimics or miR-NC. *MiR-5195-3p* upregulation suppressed luciferase activity of the wild-type but not the mutant 3′-UTR of CCNL1. Renilla luciferase activity was used as a control. **D** mRNA and **E** protein expression levels of *CCNL1* following *miR-5195-3p* mimics or miR-NC transfection. **F** *CCNL1* mRNA expression levels in 69 pairs of human PCa and matched adjacent normal tissues were measured by quantitative real-time PCR. Data are presented as mean ± standard deviation. **p < 0.01, ***p < 0.001 compared with miR-NC group. **G** *MiR-5195-3p* expression was inversely correlated with *CCNL1* miRNA expression in PCa tissues, as demonstrated by the Spearman’s correlation coefficient

To identify the potential effectors of *miR-5195-3p* in PCa progression, the target genes of *miR-5195-3p* were searched by performing bioinformatics analysis. We found that *CCNL1* 3′-UTR contains one *miR-5195-3p*-binding site and then constructed vectors containing the WT or MUT 3′-UTR of human *CCNL1* fused downstream of the firefly luciferase gene (Fig. 3A). The results from luciferase reporter assay showed that the luciferase activity was significantly decreased in both PC-3 (Fig. 3B) and DU145 (Fig. 3C) cells after co-transfection of *miR-5195-3p* mimics with *CCNL1*-WT reporters, which was abolished by the mutations in the putative *miR-5195-3p* binding site. Quantitative real-time PCR (Fig. 3D) and western blot analysis (Fig. 3E) further demonstrated that *CCNL* mRNA and protein expression levels were both significantly suppressed after *miR-5195-3p* overexpression in PC-3 and DU145 cells. In addition, the quantitative real-time PCR results showed that *CCNL1* mRNA expression was remarkably upregulated in PCa tissues compared with that in matched adjacent normal tissues (Fig. 3F), which was inversely correlated with *miR-5195-3p* expression (Fig. 3G, *r* = − 0.2387, *p* = 0.0483). Collectively, these results suggest that *miR-5195-3p* directly targeted *CCNL1* in PCa cells to downregulate *CCNL1* expression.

### *MiR-5195-3p* suppressed cell proliferation and cell cycle G1/S transition by targeting *CCNL1*

**Fig. 4:**
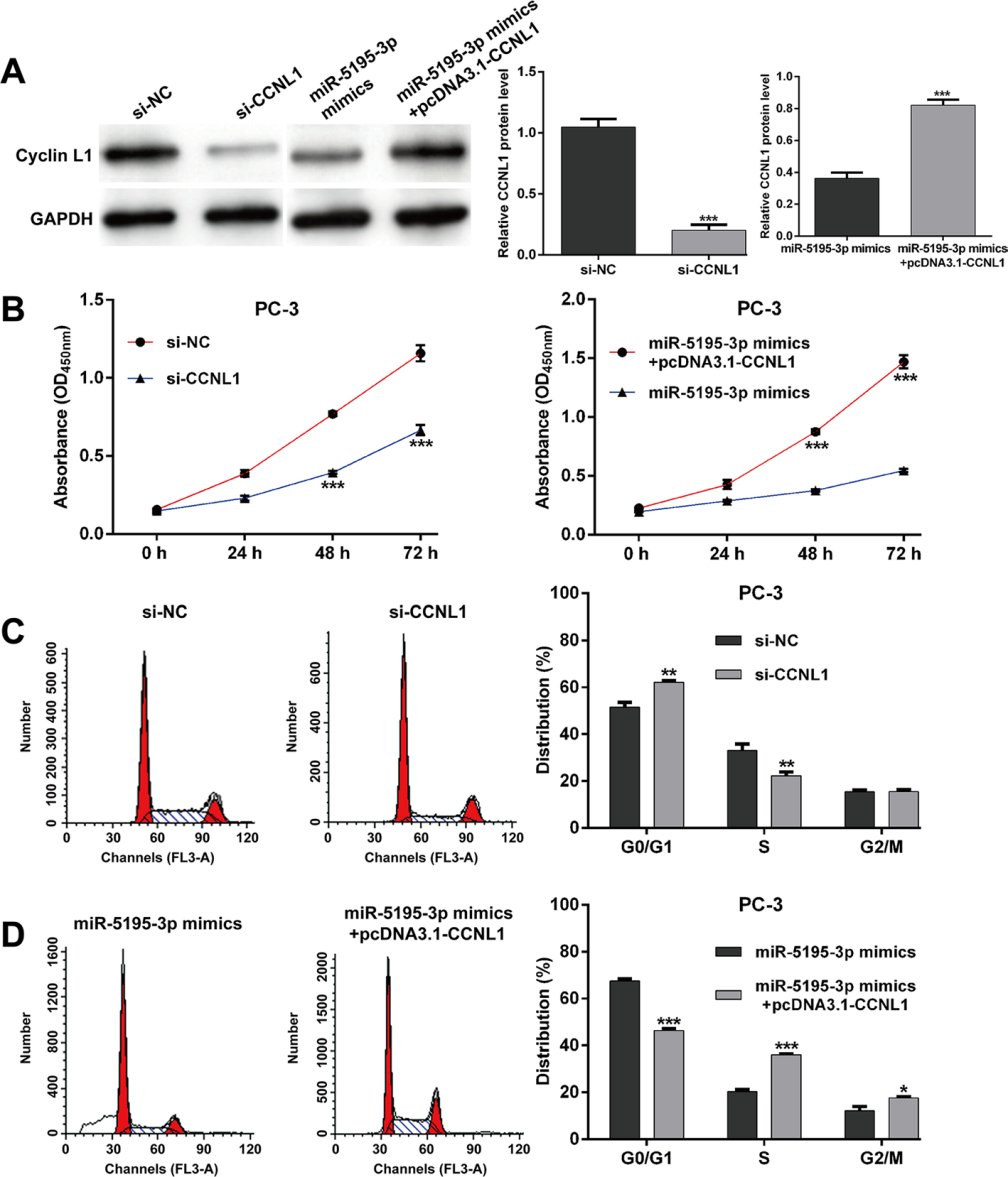
*MiR-5195-3p* suppressed cell proliferation and cell cycle G1/S transition through *CCNL1* mediation. PC-3 cells were transfected with si-CCNL1 or si-NC, as well as co-transfection with miR-5195-3p mimics and pcDNA3.1-CCNL1. **A** Western blot was used to determine the Cyclin L1 protein expression levels in the above transfected PC-3 cells. **B** Cell proliferation was tested with CCK-8 assay in the above transfected PC-3 cells. **C**–**D** Cell cycle distribution was determined by PI staining and flow cytometry analysis in the above transfected PC-3 cells. Data are presented as mean ± standard deviation. *p < 0.05, **p < 0.01, ***p < 0.001 compared with si-NC or miR-5195-3p mimics

To confirm whether *CCNL1* was the important downstream mediator involved in *miR-5195-3p* regulating PCa cell proliferation and cell cycle progression, we performed loss-of-function assays by transfection with si-CCNL1 or si-NC and rescue experiments by co-transfection with miR-5195-3p mimics and pcDNA3.1-CCNL1 in PC-3 cells. As shown in Fig. 4A, the protein expression of Cyclin L1 was obviously suppressed by si-CCNL1 transfection, which was recovered by pcDNA3.1-CCNL1 transfection in PC-3 cells. Subsequently, CCK-8 assay revealed that *CCNL1* knockdown suppressed, while overexpression promoted, the PC-3 cell proliferation (Fig. 4B). Furthermore, we found that *CCNL1* knockdown imitated (Fig. 4C), while overexpression (Fig. 4D) reversed, the effects of *miR-5195-3p* overexpression on cell cycle G1/S transition. These results suggest that *miR-5195-3p* markedly inhibited the proliferation and G1/S transition, at least partially via targeting *CCNL1* in PCa.

### *MiR-5195-3p* overexpression restricted tumor growth in vivo

**Fig. 5:**
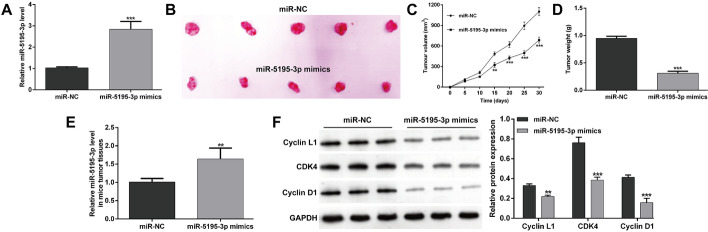
*MiR-5195-3p* induced the regression of prostate tumorigenesis in vivo. **A** *MiR-5195-3p* expression in stable *miR-5195-3p* mimics or miR-NC overexpression PC-3 cells determined by quantitative real-time PCR. **B** Image of tumor xenografts in nude mice injected subcutaneously with miR-5195-3p-overexpressing PC-3 cells. **C** Tumor volume was measured every 5 days. **D** Changes in the tumor weight in mice after *miR-5195-3p* overexpression. **E** *MiR-5195-3p* expression in tumor tissue isolated from *miR-5195-3p* mimics and miR-NC groups of nude BALB/c mice. **F** Protein expression levels of Cyclin L1, CDK4, and Cyclin D1 expression were determined by western blot analysis. Data are presented as mean ± standard deviation. **p < 0.01, ***p < 0.001 compared with miR-NC

The xenograft tumorigenicity test was performed to elucidate the in vivo suppressive potential of *miR-5195-3p*. Initially, the overexpression of *miR-5195-3p* of *miR-5195-3p* mimics in PC-3 cells was assessed and quantitative real-time PCR findings showed that *miR-5195-3p* mimics had a high overexpression efficiency (Fig. 5A). Subsequently, stably (*miR-5195-3p* mimics or miR-NC) transfected PC-3 cells were injected subcutaneously into the right flank of nude mice to produce a xenograft model of human PC-3 tumors. As shown in Fig. 5B, the tumor size was obviously smaller in *miR-5195-3p* mimics group compared with the miR-NC group in a time course of 30 days. Moreover, the time-dependent analysis illustrated that the tumor volume was significantly suppressed in mice inoculated with *miR-5195-3p* overexpressing PC-3 cells compared with the miR-NC group (Fig. 5C). Meanwhile, the tumor weight was also remarkably decreased in *miR-5195-3p* overexpressed mice (Fig. 5D). Next, we compared the relative expression of *miR-5195-3p* and found a significant high expression of *miR-5195-3p* expression in *miR-5195-3p* mimics mouse tumor tissue than in miR-NC group mouse tumor tissue (Fig. 5E). We further analyzed the protein levels of Cyclin L1, CDK4, and Cyclin D1 in tumor tissues derived from a subcutaneous xenograft murine model using western blot analysis. As shown in Fig. 5F, the protein expression levels of Cyclin L1, CDK4, and Cyclin D1 were all significantly suppressed in the *miR-5195-3p* mimics group tumor tissues relative to the miR-NC group mouse tumor tissues, which further confirmed that the upregulation of *miR-5195-3p* inhibited the growth of the prostate tumorigenesis by targeting *CCNL1*.

## Discussion

The present study revealed that *miR-5195-3p* was significantly downregulated in PCa tissues compared with adjacent normal tissues. Lower abundance of *miR-5195-3p* was associated with Gleason score, TNM stage, and worse prognosis in patients with PCa, highlighting its potential role as a tumor suppressor miRNA. The in vitro experiments demonstrated that *miR-5195-3p* overexpression reduced proliferation and induced G0/G1 cell cycle arrest in PCa cells (PC-3 and DU145). Consistent with our in vitro data, significant reduction in *miR-5195-3p* was observed in cancer samples, and the reduction was correlated with increased cell proliferation in ovarian cancer [23]. Jiang et al. [17] showed that miR-5195-3p suppressed the proliferation and invasion of human bladder cancer cells. Wang et al. [16] observed a decrease in *miR-5195-3p* expression in osteosarcoma (OS) tissues and further manifested that *miR-5195-3p* overexpression attenuated OS cell proliferation and induced apoptosis. Additionally, *miR-5195-3p* has been reported to play a suppressive role in cell growth and proliferation in glioma cells [15] and human non-small cell lung cancer cells. In particular, PC-3 and DU145 are two androgen receptor (AR)-negative PCa cell lines presenting relatively lower miR-5195-3p expression in all PCa cell lines, which were thus selected for gain-of-function assays. Based on the in vitro data, miR-5195-3p might play an important role in the development of AR-negative prostate cancer.

Furthermore, we analyzed the effects of *miR-5195-3p* overexpression on tumorigenesis and found that tumor formation in vivo was reduced with elevated *miR-5195-3p* abundance, further confirming *miR-5195-3p* functions as a tumor-suppressive miRNA in PCa. At the molecular level, we confirmed the role of *miR-5195-3p* in cell cycle G1/S transition regulation, as reflected by decreased expression of CDK4/Cyclin D1 by *miR-5195-3p* overexpression in tumor tissues. In fact, uncontrolled proliferation of tumor cells is closely associated with a deregulation of the cell cycle progression directly driven by a series of heterodimers formed by cyclins and cyclin-dependent kinases (CDKs) [24, 25]. Several lines of evidence indicate that *miR-5195-3p* is an important cell cycle regulator. For instance, *miR-5195-3p* overexpression significantly downregulated the expression levels of c-MYC and cyclin D1 but upregulated p21 expression in HCT116 cells [26]. *MiR-5195-3p* sharply suppressed the expression of its downstream promoting cell cycle regulator cyclin D1 in bladder cancer cells [17]. Thus, we speculated that *miR-5195-3p* may exert its suppressive effects on PCa cell proliferation via inducing cell cycle G0/G1 arrest through downregulating CDK4/Cyclin D1 expression.

To the best of our knowledge, several target genes of *miR-5195-3p*, including *HOXB6* in hepatocellular carcinoma [27], *MYO6* in lung cancer [14], *BIRC2* in glioma [15], *EIF4A2* in breast cancer [28], *NEDD9* in OS [16], and *KLF5* in bladder cancer [17], have been identified and confirmed, which were largely associated with aberrantly tumor cell proliferation. Here, we selected *CCNL1* as the potential target gene of *miR-5195-3p* for its role in G1/S transition [18] and carcinogenesis [19]. Furthermore, we demonstrated that *miR-5195-3p* downregulated *CCNL1* via directly binding its 3′-UTRs. The expression of *miR-5195-3p* was inversely correlated with the *CCNL1* expression level in 69 paired PCa tissues. Similarly, *CCNL1* was reported to be a direct target gene of *miR-199b-5p* and be involved in *miR-199b-5p* suppressing cell proliferation and arresting cell cycle progression in Ewing’s sarcoma [22]. As expected, our data illustrated that *CCNL1* knockdown imitated the effects of *miR-5195-3p* overexpression on PCa cell proliferation and cell cycle G1/S transition, while a converse effect was observed with *CCNL1* overexpression. Notably, it is interesting that the use of siRNA-mediated depletion of cyclinL1 does not seem to show this increase in apoptotic cells (sub-G1), while *miR-5195-3p* caused an increase in the proportion of cells at sub-G1 phase in PC-3 cells. In fact, *miR-5195-3p* induces apoptosis by directly targeting *NEDD9* in osteosarcoma [16] and targeting *EIF4A2* in breast cancer chemosensitivity [28]. These data suggest that the *miR-5195-3p* has other targets that may be primarily responsible for the apoptosis observed in PCa, which we will investigate in our next work further elucidating the molecular mechanisms underlying *miR-5195-3p* suppressing PCa cell proliferation. Our work here indicates that *CCNL1* is another identified target of *miR-5195-3p* associated with cell cycle G0/G1 phase arrest induced by *miR-5195-3p* overexpression in PCa cells. We thus speculated that *miR-5195-3p* induced cell cycle G0/G1 arrest by downregulating CDK4/Cyclin D1 via targeting *CCNL1*. In addition, our study had some limitations: (1) more sample tissues should be collected for analyzing the clinical significance of *miR-5195-3p/CCNL1* axis in PCa; (2) more PCa cell lines should be included in Figs. 4 and 5, considering the limited experimental conditions; (3) we did not investigate the effect of *miR-5195-3p* on AR-positive cell lines; (4) more targets of *miR-5195-3p* should be identified and validated; (5) other molecular mechanisms underlying the downstream pathway of *CCNL1* still need to be further explored.

## Conclusions

In summary, this study established the tumor-suppressive role of *miR-5195-3p* in PCa in vitro and in vivo. Most importantly, *CCNL1* was demonstrated to be the functionally regulated by *miR-5195-3p*, which was associated with cell cycle G1/S transition. Therefore, *miR-5195-3p* could be a potential diagnostic biomarker and therapeutic target for the treatment and early diagnosis of patients with PCa.

## Data Availability

The dataset supporting the conclusions of this article is included within the article.

## Abbreviations

PCa: Prostate cancer
*CCNL1*: Cyclin L1
SDS‐PAGE: Sodium dodecyl sulfate polyacrylamide gel electrophoresis
HR: Hazard ratio

## References

[1] Ferlay J, Soerjomataram I, Dikshit R, Eser S, Mathers C, Rebelo M (2015). Cancer incidence and mortality worldwide: sources, methods and major patterns in GLOBOCAN 2012. Int J Cancer.

[2] Miller KD, Siegel RL, Lin CC, Mariotto AB, Kramer JL, Rowland JH (2016). Cancer treatment and survivorship statistics, 2016. CA Cancer J Clin.

[3] Siegel RL, Miller KD, Jemal A (2019). Cancer statistics, 2019. CA Cancer J Clin.

[4] Shao N, Wang Y, Jiang WY, Qiao D, Zhang SG, Wu Y (2013). Immunotherapy and endothelin receptor antagonists for treatment of castration-resistant prostate cancer. Int J Cancer.

[5] Wong YN, Ferraldeschi R, Attard G, de Bono J (2014). Evolution of androgen receptor targeted therapy for advanced prostate cancer. Nat Rev Clin Oncol.

[6] Nevedomskaya E, Baumgart SJ, Haendler B (2018). Recent advances in prostate cancer treatment and drug discovery. Int J Mol Sci.

[7] Almeida MI, Reis RM, Calin GA (2011). MicroRNA history: discovery, recent applications, and next frontiers. Mutat Res.

[8] Filipowicz W, Bhattacharyya SN, Sonenberg N (2008). Mechanisms of post-transcriptional regulation by microRNAs: are the answers in sight?. Nat Rev Genet.

[9] Nam RK, Benatar T, Wallis CJD, Kobylecky E, Amemiya Y, Sherman C (2019). MicroRNA-139 is a predictor of prostate cancer recurrence and inhibits growth and migration of prostate cancer cells through cell cycle arrest and targeting IGF1R and AXL. Prostate.

[10] Ji L, Jiang X, Mao F, Tang Z, Zhong B (2019). miR-589-5p is downregulated in prostate cancer and regulates tumor cell viability and metastasis by targeting CCL-5. Mol Med Rep.

[11] Zhang Y, Zhang D, Lv J, Wang S, Zhang Q (2018). miR-410-3p promotes prostate cancer progression via regulating PTEN/AKT/mTOR signaling pathway. Biochem Biophys Res Commun.

[12] Bi CW, Zhang GY, Bai Y, Zhao B, Yang H (2019). Increased expression of miR-153 predicts poor prognosis for patients with prostate cancer. Medicine.

[13] Liu JB, Yan YJ, Shi J, Wu YB, Li YF, Dai LF (2019). Upregulation of microRNA-191 can serve as an independent prognostic marker for poor survival in prostate cancer. Medicine.

[14] Yang Q (2019). MicroRNA-5195-3p plays a suppressive role in cell proliferation, migration and invasion by targeting MYO6 in human non-small cell lung cancer. Biosci Biotechnol Biochem.

[15] Yang J, Yan DM, Xhu LX, Si DM, Liang QH (2020). MiR-5195-3p inhibits the proliferation of glioma cells by targeting BIRC2. Eur Rev Med Pharmacol Sci.

[16] Wang L, Shi G, Zhu D, Jin Y, Yang X (2019). miR-5195-3p suppresses cell proliferation and induces apoptosis by directly targeting NEDD9 in osteosarcoma. Cancer Biother Radiopharm.

[17] Jiang Z, Zhang Y, Cao R, Li L, Zhong K, Chen Q (2017). miR-5195-3p inhibits proliferation and invasion of human bladder cancer cells by directly targeting oncogene KLF5. Oncol Res.

[18] Berke JD, Sgambato V, Zhu PP, Lavoie B, Vincent M, Krause M (2001). Dopamine and glutamate induce distinct striatal splice forms of Ania-6, an RNA polymerase II-associated cyclin. Neuron.

[19] Mitra S, Mazumder Indra D, Basu PS, Mondal RK, Roy A, Roychoudhury S (2010). Amplification of cyclinL1 in uterine cervical carcinoma has prognostic implications. Mol Carcinog.

[20] Redon R, Hussenet T, Bour G, Caulee K, Jost B, Muller D (2002). Amplicon mapping and transcriptional analysis pinpoint cyclin L as a candidate oncogene in head and neck cancer. Cancer Res.

[21] Sticht C, Hofele C, Flechtenmacher C, Bosch FX, Freier K, Lichter P (2005). Amplification of Cyclin L1 is associated with lymph node metastases in head and neck squamous cell carcinoma (HNSCC). Br J Cancer.

[22] Li W, Li Y, Guo J, Pan H, Zhang Y, Wang X (2015). Overexpression of miR-199b-5p inhibits Ewing's sarcoma cell lines by targeting CCNL1. Mol Med Rep.

[23] Ebrahimi SO, Reiisi S (2019). Downregulation of miR-4443 and miR-5195-3p in ovarian cancer tissue contributes to metastasis and tumorigenesis. Arch Gynecol Obstet.

[24] Tenga MJ, Lazar IM (2013). Proteomic snapshot of breast cancer cell cycle: G1/S transition point. Proteomics.

[25] Malumbres M, Barbacid M (2009). Cell cycle, CDKs and cancer: a changing paradigm. Nat Rev Cancer.

[26] Jahangiri Moez M, Bjeije H, Soltani BM (2019). Hsa-miR-5195-3P induces downregulation of TGFβR1, TGFβR2, SMAD3 and SMAD4 supporting its tumor suppressive activity in HCT116 cells. Int J Biochem Cell Biol.

[27] Li Y, Jiang A (2020). ST8SIA6-AS1 promotes hepatocellular carcinoma by absorbing miR-5195-3p to regulate HOXB6. Cancer Biol Ther.

[28] Liu M, Gong C, Xu R, Chen Y, Wang X (2019). MicroRNA-5195-3p enhances the chemosensitivity of triple-negative breast cancer to paclitaxel by downregulating EIF4A2. Cell Mol Biol Lett.

